# Identification of Protein Hydrolysates from Sesame Meal and In Vivo Study of Their Gastric Mucosal Protective Effects

**DOI:** 10.3390/foods13244178

**Published:** 2024-12-23

**Authors:** Yutong Yuan, Xinyi Wang, Nan Ling, Jingxuan Zhou, Lei Zhao, Baoping Ji, Feng Zhou, Liang Zhao

**Affiliations:** 1 Beijing Key Laboratory of Functional Food from Plant Resources, College of Food Science and Nutritional Engineering, China Agricultural University, Beijing 100083, China; s20223061183@cau.edu.cn (Y.Y.); xaesi23@163.com (X.W.); zjx888@cau.edu.cn (J.Z.); jbp@cau.edu.cn (B.J.); 2 Nanjing Weigang Dairy Co., Ltd., No. 366 Lantian Road, Nanjing 210095, China; 801433@wgdairy.com.cn; 3 Beijing Engineering and Technology Research Center of Food Additives, School of Food and Health, Beijing Technology and Business University, Beijing 100048, China; zhaolei@th.btbu.edu.cn

**Keywords:** sesame peptides, gastric mucosal injury, LC-MS/MS, ethanol induced, Western blot

## Abstract

This study aimed to investigate the protective effects and defense mechanisms of a sesame meal protein hydrolysate against ethanol-induced acute gastric mucosal injury in mice. The target peptides in the hydrolysate were identified by LC-MS/MS, the activity was predicted by PeptideRanker, and the KM mice were orally administered distilled water, a sesame peptide, and omeprazole for 24 consecutive days. Acute gastric mucosal injury was then induced in mice with 70% ethanol, except for the CK group. The sesame peptide significantly inhibited the over-accumulation of ALT, AST, MDA, TNF-α, IL-1β, and MPO, while promoting the reduction in GSH, T-AOC, GSSG, and EGF expression. In addition, a Western blotting analysis showed that sesame peptide significantly up-regulated the expression of HO-1 and NQO1 proteins in the Nrf2/Keap1 signaling pathway, and down-regulated Keap1 protein. The defense effect of a sesame peptide on gastric mucosa may be achieved by alleviating the overproduction of lipid peroxides and improving the antioxidant activity.

## 1. Introduction

A report issued by the World Health Organization (WHO) states that over 30,000 individuals succumb annually across the globe due to alcohol misuse, underscoring that alcohol abuse has emerged as a prominent factor contributing to both illness and death [1]. Alcohol serves as an organic solvent that undergoes metabolism and absorption in the gastrointestinal (GI) tract upon ingestion. Thus, when alcohol directly contacts the gastric mucosa, it causes various metabolic and functional changes. Research has demonstrated that substantial alcohol intake may result in harm to the intestinal mucosa, culminating in an acute peptic hemorrhage, gastric ulcers, diarrhea, and other associated ailments [2,3].

Presently, the primary medications available in the market for managing gastroesophageal conditions include gastric motility modifiers, proton pump inhibitors (PPIs), and H2 receptor antagonists (H2RAs) [4]. But the results of a large number of studies point to the potential side effects of the drugs used to treat this type of disease. This can lead to nutritional deficiencies, intestinal infections, and other hazards [5]. Hence, it is essential to explore compounds that can fortify the protective function of the gastric mucosa, can enhance gastrointestinal performance, and are natural and harmless, exhibiting minimal or no detrimental effects on the human body; examples of such compounds include polysaccharides and bioactive peptides derived from food. The research findings have suggested that walnut peptides and wheat peptides possess the potential to alleviate alcohol-induced damage to the gastric mucosa [6,7].

Sesame (*Sesamum indicum* L.), an important oilseed crop, is often used as an ingredient in protein supplements due to its high protein content (22–25%) and balanced amino acid composition [8]. Sesame is usually used to extract sesame oil; in the process, it will produce a large amount of sesame meal as a by-product, and the resulting sesame meal of about more than 500,000 tons has not been put to food use [9]. Most of this is used as agricultural fertilizers or feed, with low added value. With a view to better increasing its added value, researchers usually utilize it to prepare bioactive peptides with potential functional value [10]. It has been shown that sesame peptides (SPs) have significant bioactivities and are capable of preventing diabetes and slowing down the aging process in addition to antioxidant and anti-inflammatory activities [11]. Nevertheless, it remains uncertain whether SPs have a beneficial impact on the restoration of a compromised gastric mucosal barrier.

In this investigation, we delved into the influence of SPs extracted from sesame meal on mice subjected to alcoholic gastric mucosal barrier injury, shedding light on its operational mechanism. The protective prowess of SPs against ethanol-triggered gastric mucosal injury was gauged through the assessment of parameters like the gastric mucosal damage index, damage inhibition rate, and various antioxidant indexes encompassing aspartate aminotransferase (AST), reduced glutathione (GSH), total antioxidant capacity (T-AOC), malondialdehyde (MDA), and glutathione disulphide (GSSG) levels, alongside alanine aminotransferase (ALT). Furthermore, inflammatory markers such as mouse epidermal growth factor (EGF), myeloperoxidase (MPO), mouse tumor necrosis factor-alpha (TNF-α), and mouse interleukin-1β (IL-1β) were meticulously determined, in the conclusive phase. Western blotting (WB) demonstrated that SPs regulate gastric mucosal damage through an antioxidant mechanism, and this result effectively provides a theoretical framework for the utilization of SPs for in-depth production.

## 2. Materials and Methods

### 2.1. Materials and Reagents

Sesame meal was obtained from Shijiazhuang Mingren Sesame Co., Shijiazhuang, China. Omeprazole was purchased from Renhe (Group) Development Co., Ltd., Beijing, China. ALT, AST, GSH, T-AOC, GSSG, and MDA detection kits were obtained from Beijing Solaibao Technology Co., Ltd., Beijing, China. EGF, TNF-α, MPO, and IL-1β ELISA kits were provided by Wuhan Hualianke Biological Technology Co., Ltd., Wuhan, China. BCA Protein Concentration Determination kits were purchased from Shanghai Biyuntian Biotechnology Co., Ltd., Shanghai, China.

### 2.2. Preparation of Protein from Sesame Meal

Sesame protein was extracted from sesame meal according to the method of Lu et al. [8] with modifications. The sesame powder was finished and defatted in petroleum ether and then left overnight to make the petroleum ether evaporate completely. The defatted sesame powder was mixed with water proportionally and the pH was adjusted to 11, and the supernatant was collected by centrifugation after 4 h. The pH of the supernatant was adjusted to 4.5, and the precipitate was collected by centrifugation and freeze-dried to obtain the sesame isolate protein.

### 2.3. Enzymatic Hydrolysis of Sesame Meal Protein

Sesame proteins were dispersed in water (5% *w/w*), pH adjusted to 8.5, and hydrated at 200 rpm and 45 °C for 2 h. Alkaline protease with an enzyme activity unit of 200 U/mg was used, and the main enzyme component was Bacillus licheniformis protease, which is a serine-type endoprotease, purchased from Shanghai Yuanye Biotechnology Co. The enzyme-to-substrate ratio was 1% (*w*/*w*) at 45 °C, with pH = 10.0 and a stirring speed of 200 rpm for 3 h. At the end of the digestion, the enzyme was inactivated by boiling for 10 min, and then centrifuged for 10 min at 8000 rpm. To remove salt ions from the supernatant, dialysis was carried out at 4 °C for 48 h using a dialysis bag with a molecular weight cut-off (MWCO) of 100 Da, and distilled water was changed every 8 h. Sesame hydrolyzed peptides were obtained by centrifugation under the same conditions and freeze drying.

### 2.4. Liquid Chromatograph Mass Spectrometer/Mass Spectrometer (LC-MS/MS) to Analyze Peptide Segment of SPs

An appropriate amount of a sample was dissolved in 50 mM NH4HCO3, DTT solution and was added to a final concentration of 10 mmol/L, reduced for 1 h in a water bath at 56 °C, and alkylated with 50 mM IAM for 40 min in the dark at room temperature. The peptides were desalted using a self-priming desalting column followed by vacuum centrifugation at 45 °C to remove the solvent. After reductive alkylation, the separation and identification of peptides were analyzed using LC-MS/MS (liquid chromatography–tandem mass spectrometry, Thermo Fisher Scientific, Waltham, MA, USA) equipped with an Easy-nLC 1200 system (Thermo Fisher Scientific, Waltham, MA, USA) and an Acclaim PepMap RPLC C18 column (size: 150 µm × 15 cm, particle size: 1.9 µm, Dr. Maisch GmbH, Ammerbuch, Germany). The injection volume was 5.0 µL and the flow rate was 600 nL/min. Solvent A consisted of ultrapure water with 0.1% (*v*/*v*) formic acid, while Solvent B comprised acetonitrile with 0.1% (*v*/*v*) formic acid. The solvent gradient used for SP isolation was as follows: from 4% B to 8% B in 2 min, from 8% B to 28% B in 43 min, from 28% B to 40% B in 10 min, from 40% B to 95% B in 1 min, and kept at 95% B for the last 10 min. SP sequencing was performed by Beijing Biotech Packaging (Beijing, China). Byonic was used to perform a target protein database search on the mass chromatography raw files and to sequence the peptides in the SP.

### 2.5. Animal Experimental Arrangement

**Figure 1:**
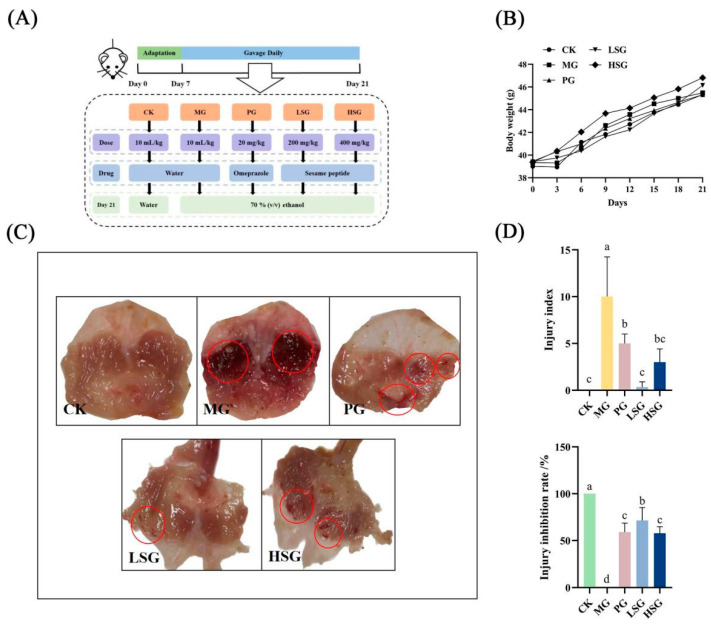
Experimental schedule of animal treatment (**A**): gastric mucosal damage was induced via ethanol in mice after gavage of different doses of sesame peptides (SPs) (200 and 400 mg/kg) for 21 days. Effect of SPs on body weight in mice (**B**); macroscopic map of mouse stomach tissue (**C**); injury index and injury inhibition rate (**D**). CK: blank control group; MG: model group; PG: omeprazole positive control group (20 mg/kg bw); LSG: low-dose sesame peptide group (200 mg/kg bw); and HSG: high-dose sesame peptide group (400 mg/kg bw). Obvious areas of damage are marked by red circles. Different letters represent significant differences between groups (*p* < 0.05).

Male Kunming mice (aged 4 weeks) were purchased from Beijing Vital River Laboratory Animal Technology Limited Company and maintained under specific pathogen-free (SPF) conditions with a 12 h/12 h 100 light/dark cycle (21 °C ± 2 °C) and a relatively constant humidity of 45% ± 10%. The mice had free access to food and water. In the experiment exploring the protective effects of SPs, the experimental procedure is shown in Figure 1A; all mice were fed a diet of standard feed for 7 days before they were divided into five groups to receive different materials via gavage (10 mL/kg of body weight), the blank control group (CK), model group (MG), positive omeprazole control group (PG, 20 mg/kg bw), low-dose sesame peptide group (LSG, 200 mg/kg bw), and high-dose sesame peptide group (HSG, 400 mg/kg bw), with 10 in each group [7]. The animal experimental procedures of this project conformed to the Guidelines for the Care and Use of Laboratory Animals developed by the National Institutes of Health (NIH), and also complied with the requirements of the Animal Ethics Committee of the Beijing Key Laboratory of Functional Foods from Plant Resources (License No. A330-2023-2). Mouse body weights were measured at fixed intervals of three days at fixed times and final body weights were weighed before the last gavage. After 21 days of gavage, mice were fasted for 24 h to ensure the complete digestion and absorption of stomach contents with free access to water. Mice in all groups except the CK were then gavaged with 70% (*v*/*v*) ethanol (10 mL/kg), and the CK was gavaged with distilled water, and euthanized 1 h later [12]. The organs (liver, kidneys, spleen, and stomach) were collected quickly after the serum was taken and dissected.

The ovary fat was removed and the organs were washed with saline and drained on filter paper and the weights were recorded. The organ index of the mice was calculated using Formula (1):

The organ index (g/g) = organ mass (g)/mouse body weight (g)(1)

### 2.6. Analysis of Gastric Mucosal Injury

Following the alcohol induction of gastric mucosal injury, we investigated the effects of the intragastric administration of SP. Stomachs were dissected along the greater curvature of the stomach to assess the severity of gastric mucosal lesions and thoroughly washed with previously cooled PBS. After taking a macroscopic unfolded view of the gastric tissue, the lesion size and area of the sample were measured. Each lesion was scored from 0 to 5 as follows: no lesion detected (a, 0 points); bleeding point/hemorrhagic point (b, 1 point); lesion less than 1 mm in length (c, 2 points); lesion 1–2 mm in length (d, 3 points); lesion 2–3 mm in length (e, 4 points); and lesion greater than 3 mm in length (f, 5 points). And the score was doubled when the lesion size exceeded 2 mm [13].

The extent of gastric injury was assessed according to Equations (2) and (3) after washing the gastric specimens as follows:

Gastric Mucosal Injury Index = Bleeding Point Score + Ulcerated Stripe Score(2)

Gastric Mucosal Injury Inhibition Rate = (Model Group Injury Index − Test Substance Group Injury Index)/Model Group Injury Index × 100%(3)

The most severely ethanol-damaged area of the gastric mucosa of each mouse was taken as about 5 mm × 5 mm, which was fixed with 4% formalin solution, conventionally gradient-dehydrated, embedded, sectioned, prepared, and stained with hematoxylin and eosin (HE) and observed under a light microscope. Partial HE staining was performed by Wuhan Safeway Biotechnology Co. Tissue damage scores based on microscopic observation ranged from 0 to 14 as follows: mucosal epithelial detachment (a, 0–3 points); upper mucosal edema (b, 0–4 points); hemorrhagic injury (c, 0–4 points); and influx of inflammatory cells (d, 0–3 points) [13]. The total score was calculated based on the above two evaluation criteria to indicate the final histopathological score.

### 2.7. Analyzing the Antioxidant Effect of SP on Ethanol-Induced Gastric Mucosal Damage and the Effect of Inflammatory Factors

The levels of MDA, T-AOC, GSH, and GSSG were used as indicators to evaluate the extent of oxidative injury in gastric tissues. These were measured according to commercial assay kits (Beijing Solepol Technology Co., Ltd., Beijing, China). MDA, T-AOC, GSH, and GSSG levels were expressed in nmol/mg prot, μmol/mg prot, μg/mg prot, and μg/mg prot, respectively.

The levels of EGF, MPO, TNF-α, IL-1β, AST, and ALT in the serum were measured using an enzyme-linked immunosorbent assay (ELISA) kit (Wuhan Hualianke Biological Technology Co., Ltd., Wuhan, China). The levels of EGF, TNF-α, and IL-1β were expressed in ng/L, and the levels of MPO were expressed in ng/mL. AST and ALT levels were quantified in U/mL.

### 2.8. Western Blotting

The total protein of gastric tissues was extracted by grinding appropriate amounts of gastric tissues in liquid nitrogen. A mixture of protease and phosphatase inhibitors (Beijing Solaibao Technology Co., Ltd., Beijing, China) and an RIPA lysis buffer (Shanghai Biyuntian Biological Technology Co., Ltd., Shanghai, China) were used to extract proteins from gastric tissues. After the completion of SDS-PAGE electrophoresis, the proteins continued to be transferred to a polyvinylidene difluoride (PVDF) membrane. The membranes were washed with Tris-buffered saline (TBST) containing Tween 20, and the primary antibodies were incubated at 4 °C overnight. Following three times of washing with TBST, the HRP secondary antibody was incubated at room temperature for 2h. The images were acquired using an Image Quant LAS 4000 Mini system (GE Healthcare, Chicago, IL, USA) and gray values were measured using Image J 2.0 software.

### 2.9. Statistical Analysis

Data were analyzed statistically using SPSS software (IBM SPSS Statistics 26) and data were expressed as the mean ± S.E.M. Using a one-way analysis of variance (ANOVA) followed by Duncan’s test for multiple comparisons, the significance level was set at *p* < 0.05. Graphs were drawn using GraphPad Prism 8 software, tables were created using Excel 2021 software, and chart summaries were drawn by Figdraw 2.0.

## 3. Results

### 3.1. Amino Acid Sequence Analysis of SPs Based on LC-MS/MS

LC-MS/MS was used to identify and analyze the amino acid sequences of the major peptides in SPs to determine the composition of the major peptides in the samples. After LC-MS/MS identification, the total ion flow chromatogram of SPs was obtained (Figure S1), and a total of 1151 peptides were detected in SPs combined with the Byonic database search. The top 50 peptides with higher scores were selected for the analysis (Table S1). The higher the score of the identified peptides, the higher the confidence of the amino acid sequence. PeptideRanker predicted the bioactivities of the selected 50 peptides, among which 10 target peptides with scores higher than 0.5 and amino acid sequences lower than 10 were selected. Table 1 shows selected peptides and their bioactivities. The molecular weight distribution of the screened peptides ranged from 328.187 to 803.464 Da with high bioactivity scores, suggesting that these target peptides have potential bioactivity. And the protein sources of the peptides were queried by searching the UniProt All Species Sequence Library (https://www.uniprot.org, 16 March 2024).

### 3.2. Effect of SPs on Body Weight and Organ Index in Mice

Within 21 days, there was a tendency for mice in the LSG and HSG to have increased body weight compared to the CK, but there was no significant difference between the groups, and the overall increase was highest in the HSG (*p* > 0.05) (Figure 1B).

The organ indexes of the groups except the liver index were not significantly different (*p* > 0.05), as shown in Table 2. In the liver index, the CK group was the lowest, the PG group was the highest, and both the LSG and HSG groups were significantly lower than the MG and PG groups (*p* < 0.05), with 38.32 mg/g and 41.81 mg/g, respectively. The gastric indexes of LSG, HSG, and PG groups were 8.31 mg/g, 9.03 mg/g, and 8.67 mg/g, respectively, which were not significantly different from those of CK and MG groups (*p* > 0.05). However, the gastric index was lowest in the CK group and highest in the MG group. Similarly, there was no significant difference (*p* > 0.05) in the kidney index and spleen index of the LSG and HSG groups compared to the CK group.

### 3.3. Apparent Damage Analysis of Gastric Tissue

We developed a model of gastric mucosal injury in KM mice, and executed the mice after gavage with 70% ethanol (0.1 mL/10 g) for 1 h to induce acute gastric mucosal injury. Figure 1C shows that the gastric mucosa of the mice in the CK group was intact, with normal color and morphology, and no obvious bleeding or ulceration was observed. On the contrary, the gastric mucosa of mice in the MG group showed obvious hemorrhagic damage with multiple hemorrhagic streaks and hemorrhagic spots, confirming the successful establishment of the gastric mucosal injury model in mice caused by ethanol. The injury inhibition rate was calculated based on the injury index obtained from the apparent injury of the gastric mucosa (Figure 1D), and there was a certain degree of an injury inhibition effect in each administered group (*p* < 0.05). It can be seen from the macroscopic map of gastric tissue that the gastric mucosal tissue damage in the PG, LSG, and HSG groups was slight compared with that in the MG group, and the gastric mucosal injury indexes were 5, 0.3, and 3, respectively, of which the gastric mucosal injury indexes in the LSG group were significantly lower than those in the other groups (*p* < 0.05). There were slightly bleeding spots in the gastric mucosa of the PG group, but no obvious large ulcers and erosions, and the degree of injury was significantly less than that of the MG group, with an inhibition rate of 58.89%. Meanwhile, the gastric mucosa of LSG and HSG groups appeared to have a small number of hemorrhagic spots after ethanol injury, but did not display apparent erosion and ulceration, with injury inhibition rates of 71.43% and 57.78%, respectively. There was no significant difference between the HSG and PG groups (*p* > 0.05), indicating that its protective effect on ethanol-induced gastric mucosal injury was similar to that of the PG group, while the injury inhibition rate of the LSG group was significantly increased by 12.54% compared with that of the PG group (*p* < 0.05), suggesting that the inhibition of ethanol-induced gastric mucosal injury was superior in the LSG group.

### 3.4. Histopathological Analysis of Gastric Tissue

**Figure 2:**
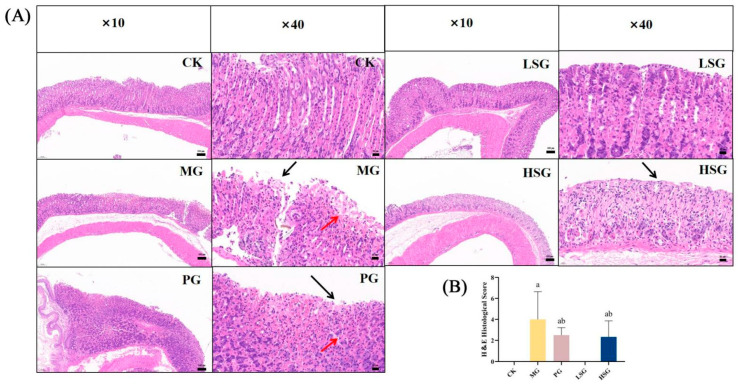
Histologic evaluation of gastric tissues for H&E staining (**A**) and histologic scoring (**B**). CK: blank control group; MG: model group; PG: omeprazole positive control group (20 mg/kg bw); LSG: low-dose sesame peptide group (200 mg/kg bw); and HSG: high-dose sesame peptide group (400 mg/kg bw). Localized epithelial cell necrosis and detachment of gastric mucosa are marked by black arrows, and localized mucosal hemorrhage is marked by red arrows. Different letters represent groups with significant differences (*p* < 0.05).

Based on HE staining and histopathological scoring (as shown in Figure 2), the epithelial glandular structure, submucosal layer, and lamina propria of the gastric mucosa of the mice in the CK were intact without obvious edematous inflammatory exudation. In the MG group, after ethanol treatment, the glandular structure of the gastric tissue was damaged, some epithelial cells were necrotic and obviously detached, and part of the mucosa was hemorrhagic. Meanwhile, its histopathological score was significantly higher than that of other groups (*p* < 0.05), further confirming the successful establishment of the ethanol-induced gastric mucosal injury model. Comparing with CK, the PG group had only a small inflammatory cell infiltration and erythrocytes in the mucosal layer. The LSG and HSG groups suffered less damage, and only HSG showed a small amount of inflammatory cell infiltration.

### 3.5. The Effects of SPs on Serum ALT and AST Levels

**Figure 3:**
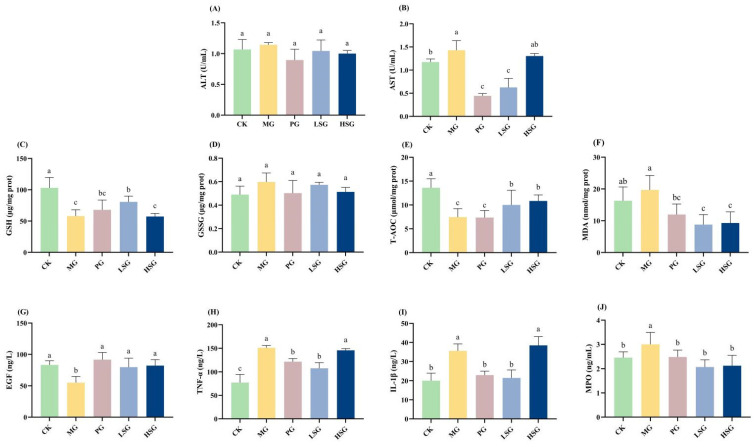
Effects of SPs on serum ALT (**A**) and AST (**B**), oxidative stress markers in gastric tissues (**C**–**F**), and serum levels of inflammatory factors (**G**–**J**) in mice. CK group: blank control group; MG group: model group; PG group: positive omeprazole control group (20 mg/kg bw); LSG group: low-dose sesame peptide group (200 mg/kg bw); HSG group: high-dose sesame peptide group (400 mg/kg bw). Different letters represent significant differences between groups at *p* < 0.05.

As shown in Figure 3A,B, serum ALT and AST were up-regulated by 0.0774 U/mL and 0.2567 U/mL in the MG group compared with the CK group, with up-regulation percentages of 26.15% (*p* > 0.05) and 17.95% (*p* < 0.05), respectively. The PG group down-regulated serum ALT, 0.248 U/mL, and down-regulated AST, 0.986 U/mL, compared to the MG group, with down-regulation percentages of 21.67% (*p* > 0.05) and 68.95% (*p* < 0.05), respectively, which confirmed the protective effect of the positive drug on the damage of the gastric mucosa associated with the alcohol delicacy. Both the LSG and HSG groups down-regulated the serum ALT values compared to the MG group, and significantly down-regulated AST values. Among them, the LSG group significantly down-regulated 0.8033 U/mL compared to the MG group, with a down-regulation percentage of 56.17% (*p* < 0.05). Moreover, the down-regulated values of ALT in the LSG and HSG groups were not significantly different from the PG group (*p* > 0.05), and the down-regulated values of AST in the LSG group were not significantly different from the PG group (*p* > 0.05).

### 3.6. The Effect of SPs in Increasing the Antioxidant Activity of the Gastric Mucosa in Mice

In the present study, the indicators related to antioxidant activity of SPs were evaluated (Figure 3C–F). Compared with the MG group, the level of GSH in the LSG group was significantly increased by 39.00% (*p* < 0.05), and the values of T-AOC in the gastric mucosal tissues of mice in the LSG and HSG groups were significantly increased by 34.14% and 45.32%, respectively (*p* < 0.05). In contrast, the GSSG values in the LSG and HSG groups were down-regulated by 0.0242 μg/mg prot and 0.0842 μg/mg prot, respectively, compared to those in the MG group, which were not significantly different (*p* > 0.05), but were likewise not significantly different from those in healthy mice in the CK group. Meanwhile, the content of MDA, a marker of oxidative stress in the gastric mucosa of mice, was detected in this study. Compared with 19.71 nmol/mg prot in the MG group, the MDA values of mice in the LSG and HSG groups were down-regulated by 10.91 nmol/mg prot and 10.21 nmol/mg prot, respectively, with a down-regulation ratio of 55.35% and 51.80%.

### 3.7. Effect of SP on Levels of Epidermal Growth Factor, Myeloperoxidase, and Pro-Inflammatory Factors

The effects of SPs on serum levels of EGF, TNF-α, MPO, and IL-1β were examined by ELISA (Figure 3G–J). EGF is considered as a gastrointestinal protective factor, which plays a crucial role in ameliorating acute gastric mucosal injury. After ethanol injury, the EGF value in the MG group was significantly reduced by 33.57% (*p* < 0.05) relative to that in the CK group, while the EGF levels in the PG, LSG, and HSG groups were not significantly different from those in the CK group (*p* > 0.05), suggesting that SPs and the positive drugs were effective in protecting the EGF levels from ethanol damage. The effect of SPs on inflammatory factors was assessed by measuring changes in relevant inflammatory cytokines in mouse serum. Relative to the CK group (77.32 ng/L), ethanol stimulation significantly increased TNF-α levels in gastric tissues of the MG, PG, LSG, and HSG groups (150.95 ng/L, 121.36 ng/L, 107.79 ng/L, and 145.61 ng/L), whereas this change was ameliorated to varying degrees in the PG and LSG groups, which resulted in their TNF-α values being down-regulated by 19.61% and 28.59% (*p* < 0.05) compared to the MG significance group. The IL-1β results of each experimental group showed that the PG and LSG groups (22.98 ng/L and 21.48 ng/L) were able to significantly reverse the decrease in IL-1β values caused by ethanol (*p* < 0.05), and their IL-1β values were not significantly different from those of the CK group (20.02 ng/L) (*p* > 0.05). MPO is an indicator of neutrophils; therefore, elevated MPO is related to the degree of gastric mucosal injury. In the MG group, after ethanol-induced acute gastric mucosal injury, neutrophils infiltrated into the damaged gastric mucosal tissues, resulting in a significant elevation in MPO activity by 22.45% compared with the normal control group (*p* < 0.05). In contrast, MPO activity in the gastric tissues of mice in the LSG and HSG groups was significantly reduced by 31.00% and 29.33% relative to the MG group (*p* < 0.05).

### 3.8. Effects of SPs on Nrf2-Mediated Antioxidant Signaling Pathways in Normal and Gastric-Ulcerated Mice

**Figure 4:**
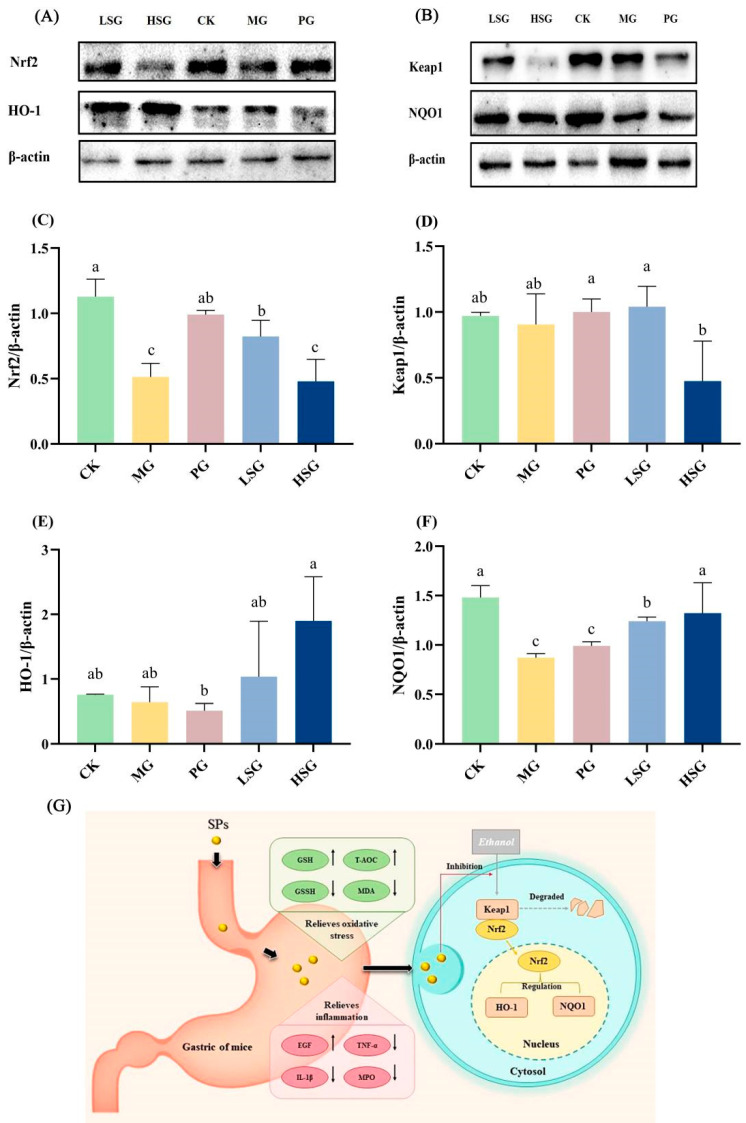
Effects of SPs on Nrf2/Keap1 antioxidant signaling pathways. Protein blot analysis of Nrf2, NQO1, Keap1, and HO-1. (**A**,**B**) Protein content analysis of Nrf2, Keap1, HO-1, and NQO1 (**C**–**F**). CK group: blank control group; MG group: model group; PG group: positive omeprazole control group (20 mg/kg bw); LSG group: low-dose sesame peptide group (200 mg/kg bw); and HSG group: high-dose sesame peptide group (400 mg/kg bw). Different letters represent significant differences between groups at *p* < 0.05. Summary of schematic diagram of SPs protecting against ethanol-induced gastric mucosal injury (**G**). SPs enhance gastric mucosal barrier by regulating antioxidant factors, inflammatory factors, and activating Nrf2 signaling pathway, thereby alleviating ethanol-induced gastric mucosal injury in mice. Black arrows represent changes in indicator levels in the control and administered groups relative to the model group.

In this study, the protein expression of Nrf2, HO-1, Keap1, and NQO1 in gastric tissues was detected by immunoblotting (Figure 4A–F). Protein blotting results showed that in the mouse stomach tissues, the Nrf2 protein expression in the LSG and PG groups was 2 times and 2.4 times higher than that in the MG group, respectively (*p* < 0.05), suggesting that the low-dose SPs as well as the positive drug significantly up-regulated the expression of Nrf2. Meanwhile, Keap1 protein expression in the HSG group was significantly down-regulated by 47.25% compared with the MG group (*p* < 0.05). To further confirm that Nrf2 is a key target of antioxidant stress, we investigated the concentrations of two key downstream target genes of Nrf2, namely HO-1 and NQO1. HO-1 protein expression in the LSG group was up-regulated by 62.5% compared with the MG group (*p* > 0.05), and was two times higher than that of the PG group (*p* < 0.05). Meanwhile, the HO-1 protein expression in the HSG group was 3 times higher than that in the MG group (*p* > 0.05) and 3.7 times higher than that in the PG group (*p* < 0.05). The NQO1 protein expression in the LSG and HSG groups was up-regulated by 42.53% and 71.26%, respectively, compared with the MG group, which were statistically significant (*p* < 0.05). The SP group also exhibited higher NQO1 protein expression content compared with the positive drug PG group (*p* < 0.05).

## 4. Discussion

Amino acid sequences are the basic unit structures that make up peptides and proteins, and different amino acids and their sequence compositions have important effects on the physicochemical properties of peptides [14,15]. Studies have shown that active peptides of different lengths are released after the peptide bond is broken, and short peptides with about 2–10 amino acids have been shown to have strong physiological and antioxidant activities [16]. SDRLFF had the highest predicted bioactivity score with hydrophobic phenylalanine at its C-terminus. The four amino acid residues of DFPAL are hydrophobic amino acids, and its C-terminal amino acid is leucine, while it has been reported that the antioxidant activity of peptides is highly correlated with the hydrophobic amino acids in their composition; particularly, when the terminal amino acid is a hydrophobic amino acid, including valine or leucine, the antioxidant activity of peptides is typically greater [17]. It is possible that this conformational relationship is attributed to the fact that the nonpolar aliphatic groups in such amino acid residues enhance the ability to trap free radicals by binding well to unsaturated fatty acids [18]. In particular, both the C- and N- terminals of APSFI and LSDFPAI are hydrophobic amino acids, conferring them potential antioxidant activity. These amino acid sequences provide an interpretable pathway for SPs to assist in the prevention of oxidative stress damage in the mouse gastric mucosa.

During the administration period, all mice were in good health, and all other groups of mice survived to the endpoint of the study, indicating that the continuous administration of SPs had no significant toxic effects on the organs of the mice. Most of the studies on ethanol-induced gastric mucosal injury have been performed on rodent models, and according to several papers and considering the differences in alcohol metabolism between rodents and humans, gastric mucosal injury generally occurs around 30 min after alcohol consumption and reaches its peak after 60 min [19,20]. Gastric mucosal injury is a multifactorial pathological process involving endogenous and exogenous factors, such as imbalance between pro-inflammatory factors (hydrochloric acid, gastric enzymes, and reactive oxygen species) and defense factors (mucus–bicarbonate barrier, mucosal blood flow, and some cytokines) [21], but among these, the effects of gastric acid and pepsin are considered to be the main cause of gastric mucosal injury. This is due to the fact that the excessive secretion of gastric acid leads to a weakening of the barrier function, which in turn leads to the self-digestion of the gastric mucosal tissue and further promotes gastric ulceration. Serum levels of alanine aminotransferase (ALT) and aspartate aminotransferase (AST) are recognized as sensitive pathological indicators of alcoholic liver dysfunction [22,23]. Since SPs were shown to inhibit the elevation of ALT and AST in ethanol-treated mice, and it was also seen that the liver coefficients of mice in the low-dose group of SPs were not significantly different from those of the CK group, this suggests that SPs have a potentially protective effect against ethanol-induced impairment of liver function.

The present study explored the effects of SPs on other indicators that indicate the extent of gastric mucosal damage, such as GSH, GSSG, T-AOC, MDA, EGF, TNF-α, IL-1β, and MPO. Oxidative stress is considered to be one of the pathogenic mechanisms of ethanol-induced gastric mucosal injury, which is attributed to the fact that the large amount of reactive oxygen species induced by ethanol promotes the production of cellular oxidants and lipid peroxides, as well as the depletion of antioxidants in the gastric mucosa, leading to cell death and apoptosis [24,25]. Therefore, increasing scavenging enzyme activities and maintaining gastric mucosal antioxidant levels are effective ways to attenuate oxidative damage and thus play an important protective role against ethanol-induced gastric ulcers. As the end product of the lipid peroxidation reaction, MDA is a key indicator to indirectly assess the level of lipid peroxidation [26]. It can be seen that SPs can improve the antioxidant activity of gastric mucosa, thus proving their application value in the prevention of alcohol-induced gastric mucosal damage.

In addition to oxidative stress, alcoholic peptic ulcers cause inflammatory pathologic changes [27], which are mainly characterized by an increased secretion of pro-inflammatory factors, such as TNF-α and IL-1β, with the suppressed production of anti-inflammatory cytokines [3,28]. A pro-inflammatory factor, TNF-α, rapidly triggers the expression of the transcription factor NF-kB, activating the release of other cytokines, thus playing an important role in inflammatory response, lipid metabolism, and apoptosis in gastric mucosal injury [29]. EGF, as a growth factor, has cytoprotective effects and facilitates mucosal blood flow and epithelial cell value addition, promoting tissue healing [7,30]. MPO levels, which are often observed in ethanol-induced gastric mucosal injury, are generated by neutrophil infiltration, which leads to the aggravation of gastric mucosal injury [13]. These results implied that SPs could effectively inhibit the alcohol-induced elevation of TNF-α, IL-1β, and MPO levels and promote the expression of beneficial EGF factors, thus preventing the excessive development of inflammation.

The Nrf2/Keap1 signaling pathway plays an important role in defense against oxidative stress in the gastrointestinal system. Nrf2 is a cellular sensor of redox status [31]. In the absence of external stress, inactive Nrf2 is anchored to Keap1 in the cytoplasm. When ethanol is ingested into the body, it is involved in oxidative metabolism that generates excessive ROS, and when stimulated externally, Nrf2 separates from Keap1 and enters into the nucleus to activate antioxidant enzymes including HO-1 and NQO1, thereby inhibiting oxidative stress and inflammation-induced gastric mucosal damage [32]. The WB results indicated that the expression of two key downstream target genes, HO-1 and NQO1, was significantly increased in the presence of SPs.

## 5. Conclusions

In conclusion, in this study, alkaline protease was used to enzymatically digest sesame seed powder proteins, and the major peptide components were analyzed by liquid chromatography–mass spectrometry and PeptideRanker, and its potential ability to reduce the damage of gastric mucous membranes by ethanol through increasing the activity of antioxidant enzymes and regulating the level of cytokines was explored. The potential therapeutic value of SPs against ethanol-induced gastric mucosal injury in mice was further confirmed by ALT, AST, GSH, GSSG, T-AOC, MDA, EGF, TNF-α, IL-1β, and MPO indexes as well as by WB experiments. This may be related to the fact that SP modulates the levels of antioxidant enzymes, inflammatory factors, and growth factors in the gastric mucosa of mice and regulates the Nrf2/Keap1 signaling pathway (Figure 4G). This result provides new methods and experimental data for utilizing sesame by-products to increase their economic value as well as exploring natural, low-cost functional foods.

## Figures and Tables

**Table 1:** Amino acid sequence, relative molecular mass, length, and bioactivity score of target peptide of SPs.

Peptide Sequence	Relative Molecular Mass (Da)	Predicted Bioactivity Score	Length	Protein Name	Intensity
SDRLFF	392.703	0.943	6	A0A6I9T2P4	317,420,000
DFPAL	562.286	0.871	5	A0A6I9TYM1	25,693,000
DFPALLK	803.464	0.772	7	A0A6I9TYM1	17,152,000
TPLFPR	730.423	0.759	6	A0A6I9US24	46,898,000
APSFI	534.293	0.746	5	A0A6I9TNX7	73,556,000
KGQTPLFPR	522.303	0.718	9	A0A6I9US24	15,441,000
GPLGPV	539.320	0.662	6	A0A6I9TFH2	59,966,000
LSDFPAI	762.402	0.618	7	A0A6I9SX06	44,146,000
VFRPH	328.187	0.614	5	A0A6I9T2I9	35,837,000
SGPKCPVTGK	515.771	0.598	10	A0A6I9SPA1	15,724,000

**Table 2:** Organ indexes of mice in each group. CK group: blank control group; MG group: model group; PG group: positive omeprazole control group (20 mg/kg bw); LSG group: low-dose sesame peptide group (200 mg/kg bw); HSG group: high-dose sesame peptide group (400 mg/kg bw). Different letters represent significant differences between groups at *p* < 0.05.

Group	Liver Index	Kidney Index	Spleen Index	Gastric Index
	(mg/g)	(mg/g)	(mg/g)	(mg/g)
CK	39.43 ± 2.32 ^c^	12.17 ± 1.41 ^a^	29.29 ± 1.50 ^a^	7.85 ± 1.82 ^a^
MG	44.68 ± 1.68 ^a^	11.95 ± 1.19 ^a^	35.28 ± 2.16 ^a^	9.36 ± 1.73 ^a^
PG	46.55 ± 3.08 ^a^	13.41 ± 2.97 ^a^	28.25 ± 2.26 ^a^	8.67 ± 2.43 ^a^
LSG	38.32 ± 2.83 ^c^	12.40 ± 1.24 ^a^	25.20 ± 0.93 ^a^	8.31 ± 1.12 ^a^
HSG	41.81 ± 1.44 ^b^	11.96 ± 0.89 ^a^	27.70 ± 1.67 ^a^	9.03 ± 1.94 ^a^

## Data Availability

The original contributions presented in the study are included in the article/Supplementary Material, further inquiries can be directed to the corresponding authors.

## Supplementary Materials

The following supporting information can be downloaded at: https://www.mdpi.com/article/10.3390/foods13244178/s1, Figure S1: Total ion flow chromatogram of SPs; Table S1: LC-MS/MS identification of SPs (top 50 scored peptides).
