# Correction: Yuan et al. Identification of Protein Hydrolysates from Sesame Meal and In Vivo Study of Their Gastric Mucosal Protective Effects. *Foods* 2024, *13,* 4178

**DOI:** 10.3390/foods14071281

**Published:** 2025-04-07

**Authors:** Yutong Yuan, Xinyi Wang, Nan Ling, Jingxuan Zhou, Lei Zhao, Baoping Ji, Feng Zhou, Liang Zhao

**Affiliations:** 1 Beijing Key Laboratory of Functional Food from Plant Resources, College of Food Science and Nutritional Engineering, China Agricultural University, Beijing 100083, China; s20223061183@cau.edu.cn (Y.Y.); xaesi23@163.com (X.W.); zjx888@cau.edu.cn (J.Z.); jbp@cau.edu.cn (B.J.); 2 Nanjing Weigang Dairy Co., Ltd., No. 366 Lantian Road, Nanjing 210095, China; 801433@wgdairy.com.cn; 3 Beijing Engineering and Technology Research Center of Food Additives, School of Food and Health, Beijing Technology and Business University, Beijing 100048, China; zhaolei@th.btbu.edu.cn

## Error in Figures

In the original publication, there were mistakes in Figures 1, 2 and 4 as published [1]. In Figure 1C, there is a problem with the control group (CK), model group (MG), and positive control group (PG) groups being mixed up in the pictures, and Figures 2A and 4A,B have the same problem, as the groups are mixed up. We revised the Hematoxylin-Eosin (H&E)-stained sections of CK, MG, and PG for Figure 2A. The Western blotting plots of Figure 4A,B were then re-replaced with the correct strip plots for all groups. The corrected Figure 1, Figure 2 and Figure 4 and their captions appear below. The Authors state that the reported data and conclusions in the published manuscript as it is online are unchanged and based on the updated Figures, and so the original scientific results conclusions are unaffected by the replacement of the Figure as per the Correction.

The authors state that the scientific conclusions are unaffected. This correction was approved by the Academic Editor. The original publication has also been updated.

## Figures and Tables

**Figure 1:**
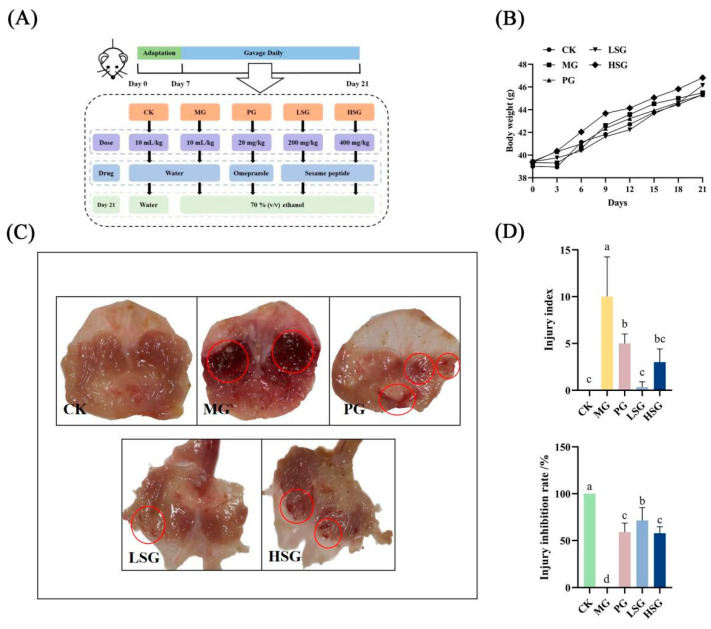
Experimental schedule of animal treatment (**A**): gastric mucosal damage was induced via ethanol in mice after gavage of different doses of sesame peptides (SPs) (200 and 400 mg/kg) for 21 days. Effect of SPs on body weight in mice (**B**); macroscopic map of mouse stomach tissue (**C**); injury index and injury inhibition rate (**D**). CK: blank control group; MG: model group; PG: omeprazole positive control group (20 mg/kg bw); LSG: low-dose sesame peptide group (200 mg/kg bw); and HSG: high-dose sesame peptide group (400 mg/kg bw). Obvious areas of damage are marked by red circles. Different letters represent significant differences between groups (*p* < 0.05).

**Figure 2:**
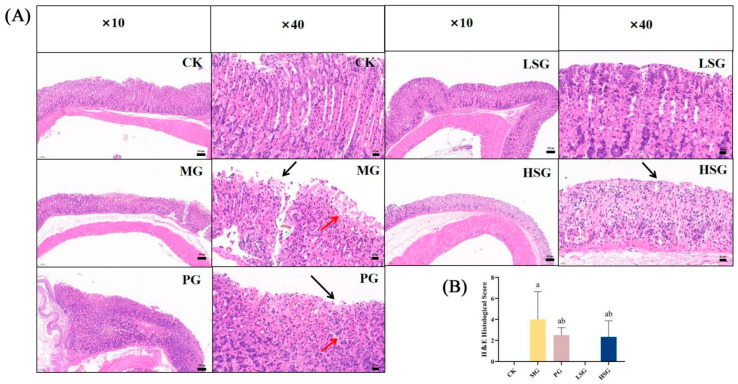
Histologic evaluation of gastric tissues for H&E staining (**A**) and histologic scoring (**B**). CK: blank control group; MG: model group; PG: omeprazole positive control group (20 mg/kg bw); LSG: low-dose sesame peptide group (200 mg/kg bw); and HSG: high-dose sesame peptide group (400 mg/kg bw). Localized epithelial cell necrosis and detachment of gastric mucosa are marked by black arrows, and localized mucosal hemorrhage is marked by red arrows. Different letters represent groups with significant differences (*p* < 0.05).

**Figure 4:**
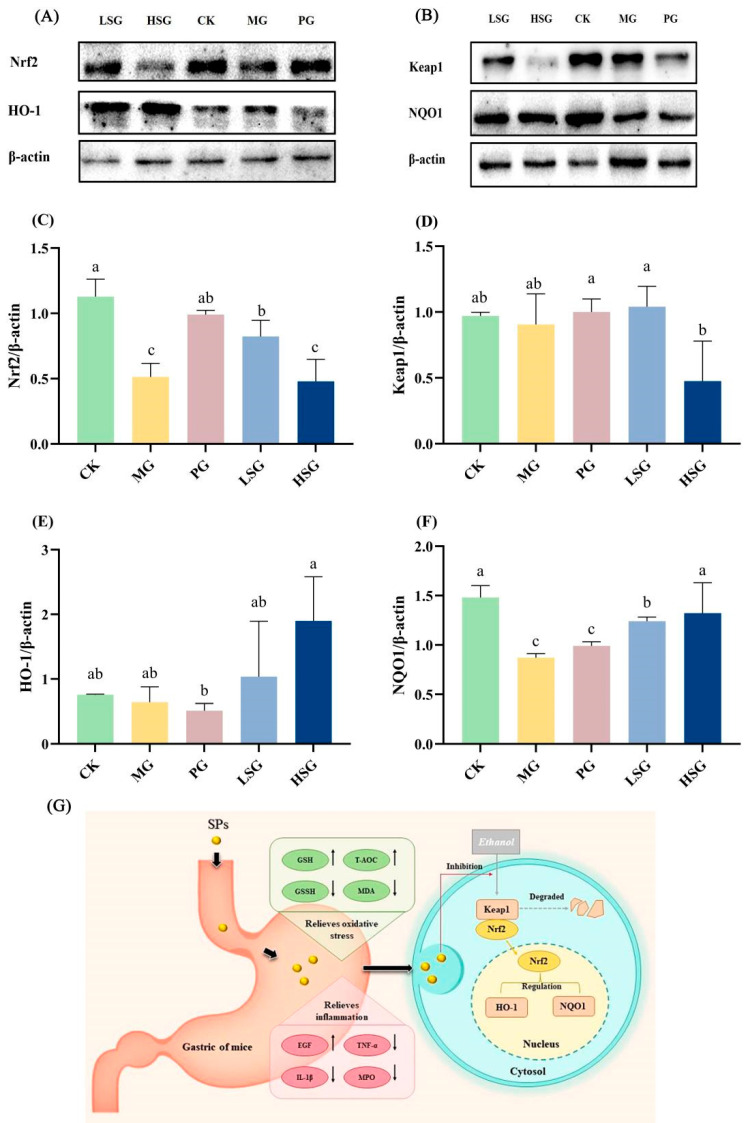
Effects of SPs on Nrf2/Keap1 antioxidant signaling pathways. Protein blot analysis of Nrf2, NQO1, Keap1, and HO-1. (**A**,**B**) Protein content analysis of Nrf2, Keap1, HO-1, and NQO1 (**C**–**F**). CK group: blank control group; MG group: model group; PG group: positive omeprazole control group (20 mg/kg bw); LSG group: low-dose sesame peptide group (200 mg/kg bw); and HSG group: high-dose sesame peptide group (400 mg/kg bw). Different letters represent significant differences between groups at *p* < 0.05. Summary of schematic diagram of SPs protecting against ethanol-induced gastric mucosal injury (**G**). SPs enhance gastric mucosal barrier by regulating antioxidant factors, inflammatory factors, and activating Nrf2 signaling pathway, thereby alleviating ethanol-induced gastric mucosal injury in mice. Black arrows represent changes in indicator levels in the control and administered groups relative to the model group.
