# Supplementary material for: Identification of Protein Hydrolysates from Sesame Meal and In Vivo Study of Their Gastric Mucosal Protective Effects
Source: Foods. 2024 Dec 23;13(24):4178. doi: 10.3390/foods13244178 (PMC11675995; doi:10.3390/foods13244178)
Supplement: Supplementary file 1 [file foods-13-04178-s001.zip › foods-3369862-supplementary.pdf]

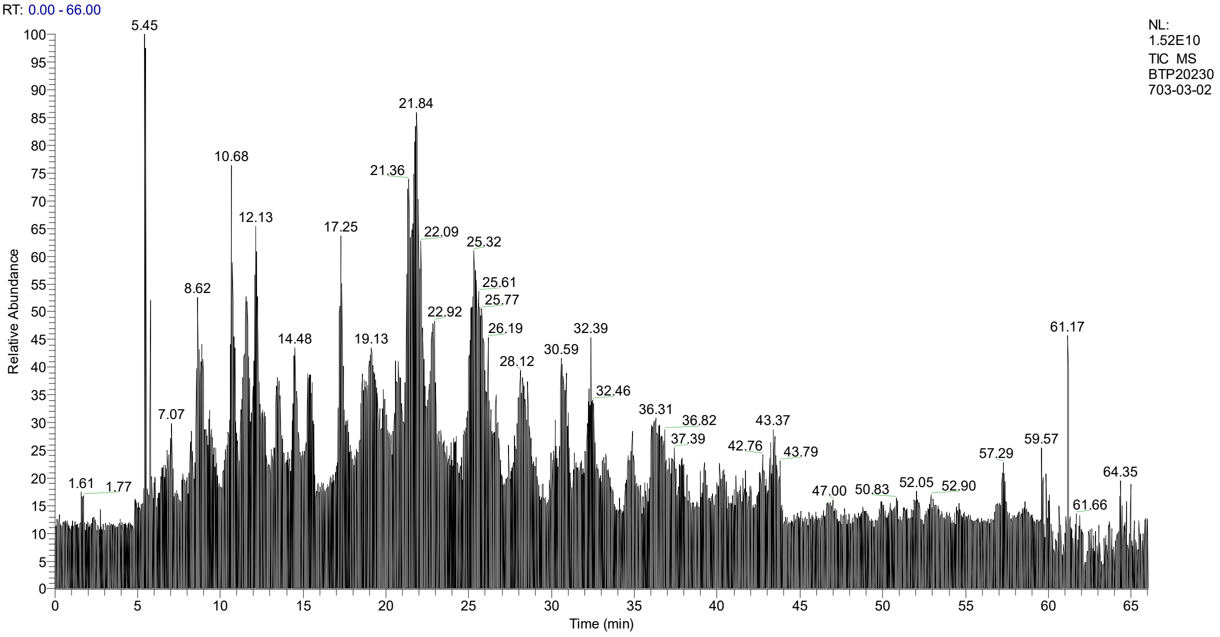

Figure S1. Total ion flow chromatogram of SPs

Table S1. LC-MS/MS identification of SPs (top 50 scored peptides)

Peptide Sequence	Relative molecular mass (Da)	Score	Scan Time	Intensity
DKIKEKLPGGH	611.351	627.5	9.0752	17382000
GPTTGDKIRL	529.303	550.1	17.2412	209010000
IDAPGHRDFIK	634.843	527.1	16.1571	12955000
GIMDKIKEKLPGGH	513.619	526.5	20.3205	55569000
SGKELEKEPKFL	468.930	507.5	14.8099	54072000
TLLIPH	693.428	501.3	25.0124	0
IKEKLPGGH	489.790	497.8	8.7884	45596000
STAGQVIRCK	581.306	495.9	16.0581	16513000
SIIDPGDSDIIK	636.837	472.8	36.19	15038000
IPTDLSLK	886.525	463.6	25.4956	18818000
LDSGISRFK	511.786	462.8	24.7257	10739000
DFPALLK	803.464	457.6	40.2176	17152000
GVPNKDDAFKTHPV	508.933	448.0	16.3868	0
TPFHPA	669.335	437.5	11.9912	39770000
TPSAKLPRIQHH	692.891	434.0	31.4378	46921000

VIDAPGHRDFIK	684.373	430.1	17.4236	18882000
NTGSPITVPVGR	599.334	420.7	24.754	86404000
TPLFPR	730.424	419.2	22.8993	46898000
SGPKCPVTGK	515.771	416.0	6.8908	15724000
ASGPKCPVTGK	551.289	409.8	7.2391	9645000
KHPEIDVPNLQ	645.346	409.5	26.6023	107410000
PALSLPN	711.402	406.5	29.1557	414690000
EIDIGVPDEVGRL	706.373	399.2	48.1531	11414000
GIGTVPVGR	428.255	399.1	17.4875	158500000
DLAPTHPIRL	378.220	395.1	28.5201	0
IEPLEKI	421.255	392.8	33.0083	28996000
SDRLFF	392.703	383.0	36.3352	317420000
KILPH	607.392	382.4	8.7517	90115000
DFPAL	562.286	382.3	43.5153	25693000
AVPVGIGI	725.454	382.1	48.5048	105170000
LVGLK	529.371	381.1	12.8198	175630000
KHPEIDVPNL	1161.624	380.5	30.0599	7322100
LSDFPAI	762.402	379.7	53.6152	44146000
VFRPH	328.188	377.4	7.9502	35837000
DALKLPTI	435.768	375.5	41.8948	306530000
TPTKQIPV	883.523	374.5	20.7702	91615000
IGTVPVGR	399.745	372.8	14.6202	0
TLPAI	514.323	372.7	32.4888	305030000
KGQTPLFPR	522.303	370.3	17.2385	15441000
PDLLIPH	804.459	369.5	41.9174	0
TVLSI	532.333	369.3	44.9333	13475000
GPKRPHDRVPL	424.582	369.1	8.7912	14561000
APSFI	534.294	366.2	35.5477	73556000
GPLGPV	539.320	365.3	23.0862	59966000
LSPLVPLK	866.569	365.1	32.0528	270420000
QRGPQERLH	560.802	364.3	7.0909	32682000
TIDIF	608.329	363.1	48.1164	39894000
ELGLLT	645.381	361.9	30.2991	36868000
TNPLTQ	673.351	361.3	11.6077	32249000
PLPVIK	666.454	361.3	32.2924	0
